# Laboratory Genetic Testing in Clinical Practice 2016

**DOI:** 10.1155/2017/5798714

**Published:** 2017-01-04

**Authors:** Ozgur Cogulu, Jacqueline Schoumans, Gokce Toruner, Urszula Demkow, Emin Karaca, Asude Alpman Durmaz

**Affiliations:** ^1^Department of Medical Genetics, Faculty of Medicine, Ege University, 35100 Izmir, Turkey; ^2^Cancer Cytogenetic Unit, Department of Medical Genetics, Lausanne University Hospital, 1011 Lausanne, Switzerland; ^3^Institute of Genomic Medicine, UMDNJ-NJ Medical School, Newark, NJ 07103, USA; ^4^Department of Laboratory Diagnostics and Clinical Immunology, Medical University of Warsaw, 00-576 Warsaw, Poland

The genetic testing in various clinical conditions emerges to have an important role in both diagnosis and treatment. Recently, a revolution in genomic technologies from the first-generation Sanger method to next-generation high throughput sequencing and microarrays has occurred. All of these technologies have been widely applied for genome, exome, transcriptome sequencing, and epigenomics. The conclusions from basic research resulted in the development of new protocols with great potential for clinical application. Selected examples of their clinical use include breakthroughs in prenatal screening, identification of rare genetic variants associated with monogenic Mendelian disorders, and efficient detection of either inherited or somatic mutations in cancer genes. This special issue is addressing laboratory genetic testing in practice, moving beyond classical concept of patient approach. Personalized medicine, which may provide accurate and effective treatment option in most of the human diseases in the future, will offer the promise of altering conventional medicine. Diagnosis, clinical findings, and treatment options vary in every individual. Thus, it is important to clarify wide spectrum of clinical and laboratory findings in examined patients. In this special issue, researchers present various cases and studies emphasizing the importance of clinical, laboratory, and genetic findings which will be beneficial in clinical practice. Among them there is ischemic stroke (IS) which is one of the important causes of morbidity and mortality. The authors showed that Vitamin K epoxide reductase complex subunit 1- (VKORC1-) 1639A allele can be a possible genetic risk factor for IS in Ukrainian population. On the other hand precision medicine mentioned above is one of the current models, which tries to explain the genetic indicators to improve the quality of medical care in recent days. Another paper points to molecular diagnostics for precision medicine in colorectal cancer (CRC). The authors discuss the future perspectives of CRC heterogeneity associated with anti-EGFR resistance and immune checkpoint blockage therapy. Authors of another paper find NGS data analysis problematic when the differentiation of the indel errors and false positive mutations is needed. The authors propose new software AGSA helping to detect false positive mutations in homopolymeric sequences at lower costs and in shorted time while increasing reliability, notably for homopolymer tracts. Tissue sampling and microdissection are very important steps in the molecular genetic analysis of cancer samples. This was described on the basis of microdissection strategy in pulmonary tumor. The authors demonstrate the importance of microdissection in morphologically different tumor components for pyrosequencing in KRAS and BRAF mutations. Despite rapid technological advances in medicine, essential hypertension (EH) etiology remains largely unknown. We publish the first research assessing the atrial natriuretic peptide gene polymorphisms in both EH and type 2 diabetes mellitus patients among Malaysian population. Copy number variations (CNVs) have attracted increasing attention as cancer susceptibility regulators. Further paper describes CNV-67048 of WW domain-containing oxidoreductase which is shown to be a risk factor of epithelial ovarian cancer in Chinese population. CNV based microarray is an important technology which provides to investigate the CNV of whole genome. The detection rate and pathogenic yield of chromosomal microarray analysis (CMA) in neurodevelopmental disorders are described. The authors established the detection rate and pathogenic yield of CMA as depending on the primary indications for testing, the age of the individuals tested, and the specialty of the ordering doctor. Targeted sequencing is the method of choice for examining genes in specific pathways or in genetic heterogeneity which is responsible for many systemic disorders. Further article describes targeted sequencing in inherited retinal dystrophies (IRD). The study demonstrates that NGS represents a comprehensive cost-effective approach for IRDs molecular diagnosis. The identification of the genetic alterations underlying the phenotype enabled the clinicians to achieve a more accurate diagnosis. The results emphasize the importance of molecular diagnosis coupled with clinic information to unravel the extensive phenotypic heterogeneity of these diseases.

*Ozgur Cogulu* *Jacqueline Schoumans* *Gokce Toruner* *Urszula Demkow* *Emin Karaca* *Asude Alpman Durmaz*

